# Hepatitis B Virus Capsid Assembly Modulators, but Not Nucleoside Analogs, Inhibit the Production of Extracellular Pregenomic RNA and Spliced RNA Variants

**DOI:** 10.1128/AAC.00680-17

**Published:** 2017-07-25

**Authors:** Angela M. Lam, Suping Ren, Christine Espiritu, Mollie Kelly, Vincent Lau, Lingjie Zheng, George D. Hartman, Osvaldo A. Flores, Klaus Klumpp

**Affiliations:** Novira Therapeutics Inc., part of the Janssen Pharmaceutical Companies, Spring House, Pennsylvania, USA

**Keywords:** HBV inhibitor, capsid assembly modulator, HBV RNA, spliced RNA, chronic hepatitis B, CHB, pgRNA

## Abstract

The hepatitis B virus (HBV) core protein serves multiple essential functions in the viral life cycle, and antiviral agents that target the core protein are being developed. Capsid assembly modulators (CAMs) are compounds that target core and misdirect capsid assembly, resulting in the suppression of HBV replication and virion production. Besides HBV DNA, circulating HBV RNA has been detected in patient serum and can be associated with the treatment response. Here we studied the effect of HBV CAMs on the production of extracellular HBV RNA using infected HepaRG cells and primary human hepatocytes. Representative compounds from the sulfonamide carboxamide and heteroaryldihydropyrimidine series of CAMs were evaluated and compared to nucleos(t)ide analogs as inhibitors of the viral polymerase. The results showed that CAMs blocked extracellular HBV RNA with efficiencies similar to those with which they blocked pregenomic RNA (pgRNA) encapsidation, HBV DNA replication, and Dane particle production. Nucleos(t)ide analogs inhibited viral replication and virion production but not encapsidation or production of extracellular HBV RNA. Profiling of HBV RNA from both culture supernatants and patient serum showed that extracellular viral RNA consisted of pgRNA and spliced pgRNA variants with an internal deletion(s) but still retained the sequences at both the 5′ and 3′ ends. Similar variants were detected in the supernatants of infected cells with and without nucleos(t)ide analog treatment. Overall, our data demonstrate that HBV CAMs represent direct antiviral agents with a profile differentiated from that of nucleos(t)ide analogs, including the inhibition of extracellular pgRNA and spliced pgRNA.

## INTRODUCTION

Hepatitis B virus (HBV) is a major cause of serious liver disease, including cirrhosis and hepatocellular carcinoma. An estimated 240 million people worldwide are affected by chronic HBV infection, and approximately 600,000 die annually from HBV-associated liver diseases (1, 2). Current approved treatment options for chronic hepatitis B (CHB) patients include nucleos(t)ide analogs and interferon alpha. Nucleos(t)ide analogs require long-term, often lifelong treatment and can select for resistant virus variants, while the treatment duration with interferon alpha is limited by its safety and tolerability profile, and such treatment shows only low rates of sustained virological response. Therefore, there is a high unmet medical need to develop options for the treatment of HBV infection with increased cure rates.

HBV is a member of the Hepadnaviridae family, and infectious HBV particles contain partially double-stranded, relaxed circular DNA (rcDNA) that can be converted into covalently closed circular DNA (cccDNA) after entry into the cell nucleus. Human RNA polymerase II mediates transcription of cccDNA, generating pregenomic RNA (pgRNA) and mRNAs for translation of viral proteins (3). The HBV core protein is encoded by the pgRNA and serves multiple functions within the viral life cycle (4). Core contains 183 to 185 amino acids with an N-terminal assembly domain and a C-terminal arginine-rich domain that interacts with nucleic acids. Oligomerization of core dimers forms icosahedral capsids, within which pgRNA and viral polymerase are encapsidated. Viral replication occurs inside capsids, where pgRNA serves as a template for the viral polymerase to synthesize HBV DNA. Capsids containing rcDNA can bind to the HBV surface proteins and be secreted as membrane-enveloped, infectious virus particles (5). In addition to facilitating viral replication and infection, core has also been implicated in cccDNA interaction and epigenetic regulation (6, 7). As there is no known human protein homolog, the HBV core protein represents a promising target for the development of antiviral molecules to treat chronic hepatitis B.

Small-molecule compounds targeting core, or capsid assembly modulators (CAMs), can be grouped into two main classes according to their effect on assembly: the phenylpropenamide and sulfamoylbenzamide chemical series accelerate formation of capsid-like particles (8, 9), while members of the heteroaryldihydropyrimidine (HAP) family of compounds induce formation of aggregated and aberrant capsid structures (10, 11). Crystal structures showed that compounds from the HAP, phenylpropenamide, and sulfamoylbenzamide series target the same hydrophobic pocket located at the core dimer-dimer interface (9, 12–14). By binding to the core protein, CAMs accelerate assembly and interfere with pgRNA encapsidation and viral replication *in vitro* in HBV-replicating cell lines (9, 11, 15–17). Compounds from the HAP series also reduced HBV viral loads *in vivo* in infected mouse models (18, 19). NVR 3-778, a first-in-class HBV CAM, demonstrated promising antiviral results in recent phase 1b studies and is being progressed into phase 2 studies for further clinical development (20).

Besides inhibiting HBV DNA replication, interference with RNA encapsidation by CAMs could have an effect on HBV RNA-containing particle production (21). The production of extracellular HBV RNA is contrary to observations from previously published studies using HBV plasmid transfection systems in Huh7 or HepG2 cell lines (5, 22) but is consistent with observations from other HBV-producing cellular systems, including hepatoma cells with stably replicating HBV and HBV-infected primary human hepatocytes (PHH) (21, 23). Circulating HBV RNA has also been detected in the serum of CHB patients, and reduction of serum HBV RNA levels in patients on pegylated interferon alpha and/or nucleos(t)ide analog therapy could be associated with higher rates of HBeAg loss, HBeAg seroconversion, or prevention of viral rebound (21, 23, 24). As different chemical classes of CAMs are currently being developed as new antiviral therapies for the treatment of chronic hepatitis B, it is important to profile their activities against HBV markers during different stages of the viral life cycle. Here, we investigated the effect of CAMs from the sulfonamide carboxamide series (represented by NVR 3-1983) and the HAP series (represented by BAY 41-4109) on the production of extracellular HBV RNA using naturally infected HepaRG cells and primary human hepatocytes. Our study further profiled the nature of extracellular HBV RNA from the supernatants of infected cells treated or not treated with nucleoside analogs and from hepatitis B virus genotype A to D-infected patient serum.

## RESULTS

### Effect of HBV CAMs on intracellular HBV RNA.

**FIG 1:**
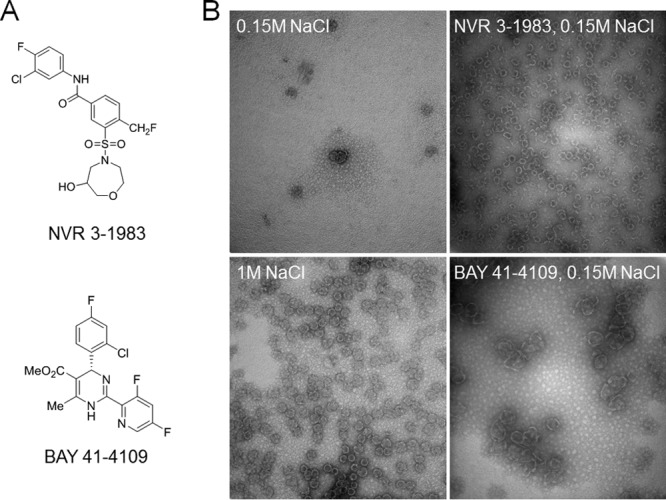
HBV capsid assembly modulators induce formation of capsid-like or aggregated particles. (A) Chemical structures of NVR 3-1983 and BAY 41-4109. (B) Electron microscopy of the HBV core protein (10 μM monomer) in the presence or absence of 10 μM NVR 3-1983 or BAY 41-4109. Core proteins remained mostly unassembled in 0.15 M NaCl and assembled into capsids at a 1 M NaCl concentration. Assembly of capsid-like particles or aggregated structures was induced by the addition of NVR 3-1983 or BAY 41-4109, respectively.

Assembly of the HBV core protein is an important step to facilitate pgRNA encapsidation and viral replication (25). If the extracellular RNA particles were produced from assembled capsids, then the assembly modulator mechanism of action would prevent the generation of such RNA particles through inhibition of RNA encapsidation. To investigate this hypothesis, we performed *in vitro* studies using NVR 3-1983 and BAY 41-4109 as representative HBV CAMs from the sulfonamide carboxamide and HAP chemical series, respectively (Fig. 1A). Electron microscopy studies showed that NVR 3-1983 induced formation of capsid-like particles from recombinant core dimers (Fig. 1B). In contrast, BAY 41-4109 induced formation of aggregated and misassembled capsid structures, consistent with previously reported data for compounds from the HAP series (10, 11). These two compounds thus represent CAMs that accelerate formation of either capsid-like particles or aberrant structures.

**FIG 2:**
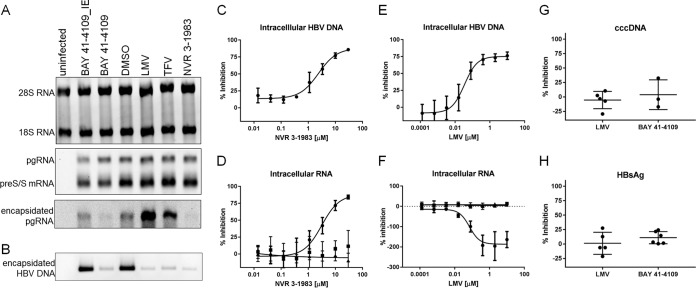
Effect of CAMs and nucleos(t)ide analogs on intracellular HBV RNA and DNA. (A and B) Uninfected and infected HepaRG cells were treated with DMSO or 30 μM BAY 41-4109_IE, BAY 41-4109, LMV, TFV, or NVR 3-1983 for 6 days prior to Northern blot analysis of intracellular ribosomal 18S and 28S RNA (A, top), HBV pgRNA and preS/S mRNA transcripts (A, middle), and encapsidated pgRNA (A, bottom) and Southern blot analysis of intracellular encapsidated HBV DNA (B). (C to F) Infected HepaRG cells were treated with increasing concentrations of NVR 3-1983 or LMV for 6 days, prior to measuring the amonts of intracellular HBV DNA (C, E) or encapsidated HBV RNA (●), total HBV mRNA transcripts (■), and beta-actin RNA (▲) (D, F). (G and H) Effect of 10 μM LMV or BAY 41-4109 on HBV cccDNA (G) and secreted HBsAg (H). Data points and error bars represent means and standard deviations from at least three independent studies, respectively.

The effect of NVR 3-1983 and BAY 41-4109 on HBV RNA was examined using HepaRG cells infected with HBV. Similar to primary human hepatocytes, differentiated HepaRG cells are permissive to HBV infection and viral replication is dependent on cccDNA establishment and maintenance (26). In addition to HBV CAMs, persistently infected HepaRG cells were treated with lamivudine (LMV) and tenofovir (TFV) as representative nucleos(t)ide analogs targeting the viral polymerase. Results from Northern blot analysis showed that host rRNA (18S and 28S rRNAs) and viral mRNA transcripts (pgRNA and preS/S mRNAs) were not affected by either CAMs or nucleos(t)ide analogs (Fig. 2A, top and middle). However, encapsidation of pgRNA was inhibited by NVR 3-1983 and BAY 41-4109, while higher levels of encapsidated pgRNA were detected in cells treated with the two nucleos(t)ide analogs (Fig. 2A, bottom). Infected cells treated with BAY 41-4109_IE, the inactive enantiomer of BAY 41-4109, showed similar levels of encapsidated pgRNA as the dimethyl sulfoxide (DMSO)-treated control (Fig. 2A, bottom). Viral replication was suppressed by both CAMs and nucleos(t)ide analogs, as confirmed by Southern blotting, showing a reduced amount of intracellular encapsidated HBV DNA compared to that from untreated cells (Fig. 2B). Titration studies showed that NVR 3-1983 inhibited intracellular encapsidated HBV DNA and RNA with mean 50% effective concentrations (EC_50_s) of 2.4 and 3.5 μM, respectively (Fig. 2C and D; Table 1). BAY 41-4109 also inhibited intracellular encapsidated HBV DNA and RNA with EC_50_s of 0.33 and 0.27 μM, respectively (Table 1). LMV inhibited HBV DNA replication (Fig. 2E), but the level of intracellular encapsidated HBV RNA actually increased in a dose-dependent manner (Fig. 2F; Table 1). The antiviral effect of CAMs or nucle(t)oside analog treatment was not associated with cytotoxicity, as similar levels of intracellular beta-actin RNA were observed up to the highest concentration tested (Fig. 2D and F; Table 1). We also observed that in persistently infected HepaRG cells, neither CAM nor the nucleoside analogs reduced cccDNA levels (Fig. 2G), consistent with the absence of inhibition of total intracellular HBV mRNA (Fig. 2A, D, and F) and HBsAg (Fig. 2H) production.

**TABLE 1:** Antiviral activities of NVR 3-1983, BAY 41-4109, and LMV against HBV in persistently infected HepaRG cells

Compound	Mean ± SD (range) EC_50_^*a*^ (μM)						
	Intracellular		Intracellular encapsidated		Extracellular		
	HBV RNA	Beta-actin RNA	HBV DNA	HBV RNA	HBV DNA	HBV RNA	HBsAg
NVR 3-1983	>30	>30	2.4 ± 1.3 (1.0–3.3)	3.5 ± 1.7 (1.6–4.9)	1.9 ± 0.58 (1.2–2.7)	1.9 ± 1.0 (0.71–3.0)	>30
BAY 41-4109	>10	>10	0.33 ± 0.053 (0.25–0.37)	0.27 ± 0.092 (0.14–0.36)	0.25 ± 0.067 (0.17–0.34)	0.35 ± 0.16 (0.21–0.52)	>10
LMV	>10	>10	0.033 ± 0.014 (0.016–0.048)	ND^*b*^	0.017 ± 0.001 (0.016–0.018)	ND	>10

a Data are from at least three independent studies.

b ND, not determined, as no inhibition was observed.

### Effect of HBV CAM on extracellular HBV RNA.

**FIG 3:**
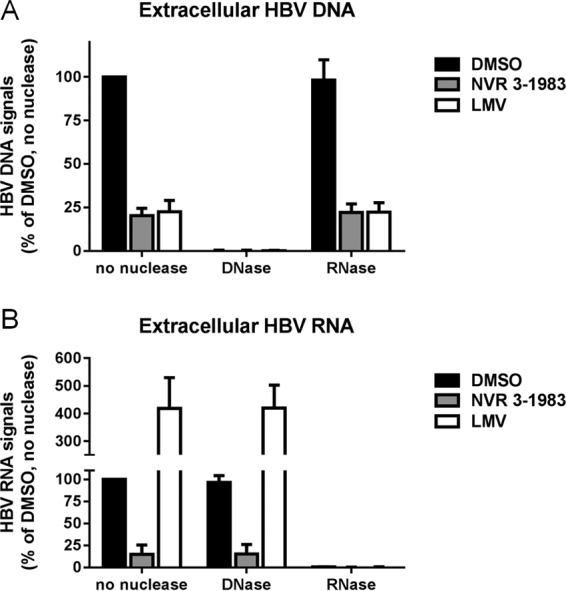
Effect of nuclease treatment on extracted HBV DNA and RNA from the supernatant of infected HepaRG cells treated with HBV inhibitors. Cells were incubated with DMSO or 30 μM NVR 3-1983 or LMV for 6 days. HBV DNA and RNA from culture supernatants were extracted and treated with DNase or RNase, prior to quantitative assays. Extracellular HBV DNA (A) and HBV RNA (B) levels were normalized to those for DMSO-treated samples without nuclease treatment. Results and error bars represented means and standard deviations from at least three independent experiments, respectively.

In order to determine if the suppression of intracellular pgRNA encapsidation by CAMs could reduce the production of extracellular HBV RNA, we next monitored the levels of extracellular HBV DNA and HBV RNA from the supernatants of infected cells treated or not treated with HBV inhibitors. HBV DNA was suppressed by both NVR 3-1983 and LMV (Fig. 3A), but only NVR 3-1983 reduced the level of extracellular HBV RNA (Fig. 3B). In order to confirm that the quantitative signals were selectively measuring RNA or DNA, nucleic acids from the supernatants were extracted and subjected to either DNase or RNase digestion. The HBV DNA signals from the DMSO-treated control and compound-treated samples were highly sensitive to DNase, as a >99% signal reduction was observed with DNase treatment, while similar levels of DNA signals were detected in the presence of RNase (Fig. 3A). Monitoring of HBV RNA showed that the RNA signals from untreated and compound-treated samples were resistant to DNase but were sensitive to RNase: RNase degraded >99% of the signals, including the increased RNA signals, when cells were treated with LMV (Fig. 3B).

**FIG 4:**
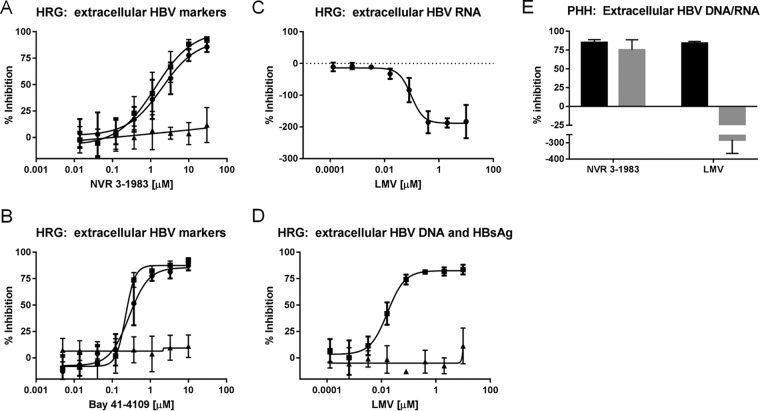
Effect of inhibitors against extracellular HBV DNA, HBV RNA, and HBsAg. Infected HepaRG cells (HRG) were treated with increasing concentrations of NVR 3-1983 (A), BAY 41-4109 (B), or LMV (C, D) for 6 days. The levels of HBV RNA (●), HBV DNA (■), and HBsAg (▲) from the cell culture supernatants were measured. (E) Primary human hepatocytes (PHH) infected with HBV were treated with either 30 μM NVR 3-1983 or 10 μM LMV for 6 days prior to determining the levels of extracellular HBV DNA (black bars) and HBV RNA (gray bars). HBV DNA and RNA levels were monitored by branched DNA and QuantiGene assays, respectively. HBsAg was measured by enzyme-linked immunosorbent assay. Data points and error bars represent means and standard deviations from at least three independent studies, respectively.

The efficiencies of inhibition of extracellular HBV RNA by NVR 3-1983 (EC_50_ = 1.9 μM) and BAY 41-4109 (EC_50_ = 0.35 μM) (Fig. 4A and B) were very similar to the efficiencies of inhibition of intracellular HBV RNA encapsidation, HBV DNA replication, and virion production (Table 1). On the contrary, LMV increased the levels of extracellular HBV RNA in a dose-dependent manner (Fig. 4C), while it maintained its activity against HBV virion production (Fig. 4D). The antiviral effect of NVR 3-1983 and LMV was also evaluated in primary human hepatocytes (PHH) infected with HBV. Results showed that infected PHH responded to HBV CAM and nucleoside analog treatment in a similar manner as infected HepaRG cells: HBV DNA replication was inhibited by both NVR 3-1983 and LMV, while extracellular HBV RNA was suppressed by NVR 3-1983, but not LMV (Fig. 4E). Taken together, these data confirmed that CAMs suppressed the production of extracellular HBV DNA and RNA, while nucleoside analogs inhibited HBV DNA but not extracellular HBV RNA.

### Profiling of extracellular HBV RNA.

**FIG 5:**
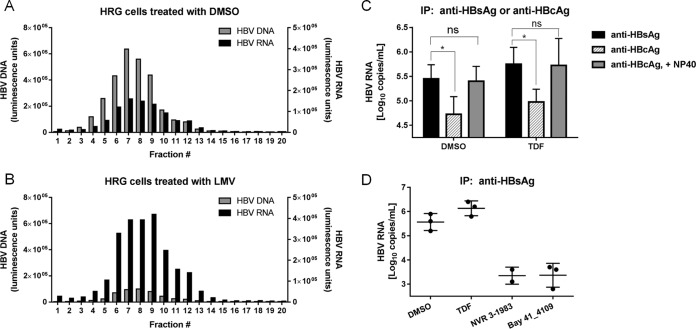
Profiling of extracellular HBV RNA-containing particles. The migration of HBV RNA-containing particles on an OptiPrep density gradient (10% to 50%) was compared to that of Dane particles. Supernatants were collected from HBV-infected HepaRG cells (HRG) treated with DMSO (A) or 30 μM LMV (B) and concentrated. The levels of HBV DNA or HBV RNA from each fraction were determined. (C) HBV RNA-containing particles from the supernatants of DMSO- or TDF-treated cells were immunoprecipitated with anti-HBsAg or anti-HBcAg antibodies. Immunoprecipitation (IP) with anti-HBsAg and anti-HBcAg antibodies was performed in the absence of detergent. Immunoprecipitation with anti-HBcAg antibodies was also performed in the presence of 1% NP-40. (D) HBV RNA-containing particles from the supernatants of infected HepaRG cells treated with HBV inhibitors were immunoprecipitated with anti-HBsAg antibodies. Data points and error bars represent means and standard deviations from at least three independent studies, respectively. ns, not significant; *, *P* < 0.05.

By misdirecting assembly, CAMs reduced pgRNA encapsidation and HBV RNA secretion, suggesting that extracellular HBV RNA was associated with assembled capsids. We performed OptiPrep gradient centrifugation and immunoprecipitation studies using supernatants from both DMSO- and LMV-treated cells in order to investigate if extracellular HBV RNA existed within virion-like particles. Results from the fractionation studies showed HBV RNA comigrating with HBV DNA, suggesting that HBV RNA-containing particles were similar in density to Dane particles (Fig. 5A and B). Peak fractions from the LMV-treated samples showed a 10-fold reduction in HBV DNA signals compared to those for the DMSO-treated control, but the signals for fractions containing HBV RNA were increased by about 3-fold (Fig. 5B). By immunoprecipitation, we detected the presence of HBV enveloped proteins, as anti-HBsAg antibodies precipitated HBV RNA particles from the supernatants of both untreated and tenofovir disoproxil fumarate (TDF)-treated cells (Fig. 5C and D). The levels of HBV RNA particles precipitated by the anti-HBsAg antibodies declined by more than 90% upon treatment with NVR 3-1983 and BAY 41-4109 (Fig. 5D). Replacement of anti-HBsAg antibodies by anti-HBcAg antibodies resulted in a 10-fold reduction in the amount of immunoprecipitated HBV RNA-associated signals in both DMSO- and TDF-treated culture supernatants. However, upon detergent treatment, the levels of HBV RNA precipitated by anti-HBcAg were similar to those obtained with anti-HBsAg, indicating that secreted RNA was contained within capsids and not just inside subviral particles. These data indicate that interfering with capsid assembly by CAMs reduces the level of production of HBV RNA-containing particles and that the majority of extracellular HBV RNA is contained within enveloped and encapsidated particles.

### Extracellular HBV RNA comprises pgRNA and spliced RNA variants.

**FIG 6:**
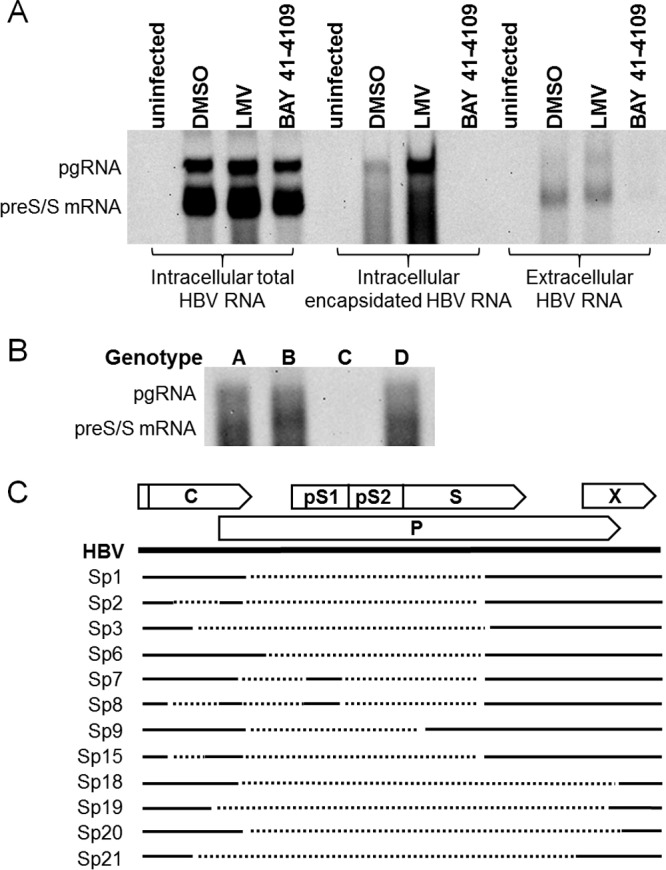
Extracellular HBV RNA contains pgRNA and spliced RNA variants. (A) Northern blot analysis of intracellular and extracellular HBV RNA from uninfected and infected HepaRG cells treated with DMSO, LMV, or BAY 41-4109. (B) Northern blot analysis of HBV RNA from representative patient samples infected with genotype A, B, C, or D HBV. (C) Location of spliced introns relative to the HBV genome (solid line). The arrows at the top indicate the open reading frames of HBV. Dotted lines below indicate spliced introns from sequenced clones generated using the supernatants of infected HepaRG cells or patient serum. The spliced variant (Sp) numbering is according to previous publications (29, 30).

The nature of extracellular HBV RNA was examined by Northern blotting using concentrated supernatants from uninfected, infected, LMV-treated, or BAY 41-4109-treated cells. Extracellular RNA was analyzed alongside intracellular HBV RNA and encapsidated pgRNA in order to compare the size and migration patterns of the transcripts (Fig. 6A). As expected, LMV and BAY 41-4109 did not affect intracellular pgRNA or preS/S mRNA transcription (Fig. 6A, left), while intracellular pgRNA encapsidation was inhibited by BAY 41-4109 but not LMV (Fig. 6A, middle). Extracellular HBV RNA was detectable from the supernatants of both untreated and LMV-treated samples but not from the supernatants of cells treated with BAY 41-4109 (Fig. 6A, right). Unexpectedly, the extracellular HBV RNA appeared to consist of two populations: a fainter but visible upper band with a size consistent with that of pgRNA and a more intense lower band with a size similar to that of the preS/S mRNA. Northern blot analysis of patient serum samples also showed a similar profile: a faster-migrating band that appeared to be more intense than the pgRNA band (Fig. 6B). We were unable to detect HBV RNA from one of the four patient serum samples due to a relatively small amount of HBV RNA (which was about 7-fold lower than that in the other three patient samples).

Previous studies profiling extracellular HBV RNA by real-time PCR-based methods using primers that hybridized specifically to pgRNA indicated that the extracellular HBV RNA was pgRNA (21). However, the appearance of the more intense lower band similar in size to that of the preS/S transcripts suggested that other HBV RNA forms could be encapsidated and secreted. Since extracellular HBV RNA existed within capsids, we hypothesized that it should retain the 5′-epsilon sequence for signaling encapsidation (27, 28). To determine the genomic identity of extracellular HBV RNA, RNA from the supernatants of DMSO- or LMV-treated cells was reversed transcribed using a 3′ rapid amplification of cDNA ends (RACE)-based primer that hybridized to the poly(A) region of mature HBV RNA transcripts (24), followed by amplification and sequencing of amplicons using primers that framed the 5′ and 3′ termini of the viral genome. Unexpectedly, the cDNA templates did not generate pgRNA-sized amplicons but, rather, generated those that were ≤2 kb in size. A total of 16 different clones from the supernatant of DMSO-treated cells and 12 different clones from the supernatant of LMV-treated cells were sequenced (Table 2). Comparison of these sequences with the HBV genome sequence indicated that while these amplicons retained the 5′-epsilon sequence and the 5′ and 3′ ends of pgRNA, they also contained an internal deletion(s) that ranged from 282 to more than 2,000 bp in length (Table 2). Most of these pgRNA variants contained singly spliced introns (spliced variant 1 [Sp1], Sp3, Sp6, Sp18 to Sp21), while one variant (Sp7) was doubly spliced. Notably, spliced pgRNA variants were present in the supernatants of both DMSO- and LMV-treated cells. In particular, 9 out of the 12 clones sequenced from the LMV-treated samples were identical to those from the DMSO-treated samples, as represented by Sp1, Sp3, and Sp6 (Table 2).

**TABLE 2:** HBV RNA spliced variants detected in supernatants of infected HepaRG cells treated with and without LMV, as well as patient sera infected with HBV of genotypes A to D

Spliced variant [size of deletion(s) (bp)]	No. of sequenced clones containing spliced variants^*a*^							Reference(s)^*b*^
	HRG-S		Patient samples				Total	
	DMSO	LMV	3.4	3.7	3.56	3.57		
Sp1 (1,223)	3	3	3	4	5	8	26	29–34, 41
Sp2 (282, 1,223)	0	0	0	4	0	0	4	29, 30
Sp3 (1,602)	5	5	8	0	5	0	23	29–31
Sp6 (1,199)	2	1	0	0	0	0	3	29–31, 34
Sp7 (455, 684)	1	0	0	0	0	0	1	29, 30
Sp8 (282, 454, 688)	0	0	0	3	0	0	3	29, 30
Sp9 (1,016)	0	0	0	0	0	3	3	29, 30
Sp15 (168, 1,223)	0	0	0	0	1	0	1	29
Sp18 (2,266)	2	0	0	0	0	0	2	
Sp19 (2,179)	2	0	0	0	0	0	2	
Sp20 (2,290)	1	0	0	0	0	0	1	
Sp21 (2,422)	0	3	0	0	0	0	3	
Total no. of clones sequenced	16	12	11	11	11	11	72	

a Variants in HepaRG cell supernatants were sequenced from 1.8- and 0.7-kb amplicons; variants in patient samples were sequenced from 1-, 1.5-, and 2-kb amplicons. HepaRG cell samples (HRG-S) were infected with genotype D HBV; patient samples 3.4, 3.7, 3.56, and 3.57 were infected with genotype B, C, D, and A HBV, respectively.

b The reference(s) for the previously published HBV RNA or DNA variants.

In order to determine if extracellular spliced RNA also exists in patient samples, HBV RNA from patient serum samples infected with HBV strains representing genotypes A to D were reverse transcribed, and cDNA templates were amplified and sequenced. Similar to the amplicons from infected culture supernatants, the amplicons from patient samples also contained less than 2-kb-sized genomes (Table 2). Among these variants, Sp1 and Sp3 were the most abundant, with the Sp1 variant being detectable across all samples. Sp2, Sp8, Sp9, and Sp15 were variants detected in patient samples but not in cell culture supernatants. Similar to the spliced pgRNA from the culture supernatants, the majority of the variants found in patient sera were singly spliced, but variants containing two or three spliced introns were also observed. In total, we identified 12 different spliced variants from the infected culture supernatants and patient serum samples (Fig. 6C). Eight of the 12 have previously been reported to be either RNA or DNA splice variants (29–35), while 4 (Sp18 to Sp21) were new variants. Most of the internal deletions initiated at the 3′ end of the core gene and continued to the middle of the HBsAg gene. About half of the spliced variants retained most of the precore/core gene, and 75% of the spliced variants retained the HBx gene.

Since a faint pgRNA band was detected in the Northern blot experiments but only spliced variants were identified by sequencing, it is possible that pgRNA was missed during cDNA amplification. In order to detect pgRNA, primers were designed to target within the most common spliced intron region. Using this strategy, the spliced products would not be amplified, as the internal primers could anneal only to the unspliced genome. We were indeed able to amplify sequences that indicated the presence of pgRNA both in infected HepaRG culture supernatants and in all four of the patient serum samples infected with HBV strains of genotypes A to D.

## DISCUSSION

The HBV core protein is a promising antiviral target for the treatment of hepatitis B, and small-molecule compounds targeting core are currently being developed as a new class of direct-acting antiviral agents. High-resolution crystal structures are now available to decipher the molecular interaction between the HBV core protein and CAMs from different chemical series (12–14), which disrupt the viral life cycle by accelerating and misdirecting capsid assembly. Here, using NVR 3-1983 and BAY 4109 as representative CAMs that induce formation of capsid-like and misassembled particles, respectively, the results of a systematic analysis of intracellular and extracellular HBV RNA support the hypothesis that the misdirection of capsid assembly by CAMs inhibits HBV RNA encapsidation and in turn prevents the production of HBV RNA-containing particles. Our data demonstrated that CAMs inhibited pgRNA encapsidation, HBV DNA replication, and virion and HBV RNA particle production with similar efficiencies, indicating that CAMs could directly affect multiple steps of the viral life cycle by targeting core. On the contrary, nucleos(t)ide analogs suppressed only HBV DNA replication but did not block pgRNA encapsidation or HBV RNA particle production. Consistent with their respective targets and mechanisms of action, neither CAMs nor nucleos(t)ide analogs reduced the level of cccDNA transcription or HBsAg production in persistently infected hepatic cells.

Although clinical studies monitoring serum HBV RNA have suggested that this marker may be a predictor of the treatment response and cccDNA activity (21, 23, 24, 36–39), more data will be needed to confirm the therapeutic significance and to better understand the potential biological function of extracellular HBV RNA. Here, we characterized the HBV RNA produced from infected HepaRG cells before and after nucleos(t)ide analog treatment and also from the serum of patients infected with HBV strains representing genotypes A to D. Unexpectedly, besides pgRNA, a substantial amount of spliced pgRNA variants was detected in both the culture supernatants and the patient serum samples. Sequencing analysis showed that these variants retained the 5′ and 3′ ends of pgRNA but also contained single or multiple internal deletions of various lengths. The presence of the epsilon element at the 5′ terminus would enable packaging of these spliced variant into capsids, as supported by the immunoprecipitation data indicating that extracellular HBV RNA particles were precipitated by anti-HBcAg antibodies after detergent treatment and by anti-HBsAg antibodies prior to envelope removal. Although envelopment of encapsidated HBV RNA has not been detected previously from HepG2 or Huh7 hepatoma cells transfected with HBV DNA plasmids (5, 22), this observation is consistent with observations from other HBV-producing cellular systems, including primary human hepatocytes (21, 23). Indeed, the sequences of some of these extracellular pgRNA spliced variants were identical to those of previously published spliced viral transcripts found inside infected patient livers (29, 40, 41). Notably, most of the variants from the supernatants of LMV-treated cells were also detected in untreated cells, indicating that splicing was not associated with inhibition of the viral polymerase. Besides spliced variants, other forms of HBV RNA have been reported; for example, it was observed that nucleos(t)ide analog treatment could result in the accumulation of intracellular truncated hepadnaviral RNA in chicken hepatoma and HepAD38 cells (42). In our analysis of intracellular HBV RNA, however, such a truncated form was not detected in infected cells treated or not treated with LMV. It is possible that the posttranscriptional modification of HBV RNA was different between transfected hepatoma cell lines and infected hepatocytes, which are differentiated cells that support cccDNA-dependent HBV replication. On the other hand, profiling of extracellular HBV RNA by real time-PCR from other studies described the existence of full-length and truncated pgRNA transcripts, based on the amplification of pgRNA sequences either at the polyadenylated site or at an alternative polyadenylated site found within the HBx coding region, respectively (24, 43). As the spliced variants retained sequences from both ends of pgRNA, our data are not in disagreement with these pgRNA forms. However, PCR amplification can be limiting for genome analysis because it amplifies only short fragments of the viral genome. Considering the diversity, a more robust RNA sequencing technology would be useful in further deciphering the heterogeneity of secreted HBV RNA, especially since the amplicons sequenced here are limited to clonal isolates. Interestingly, the majority of our spliced variants retained the HBx gene, which coincided with the transcriptome sequencing data from a recent report mapping most viral reads to the HBx transcript in both culture supernatants and patient plasma (44). It is anticipated that the functional roles of extracellular HBV RNA will be the subject of further investigation, as HBx is a protein essential for the maintenance of cccDNA transcription (45, 46).

It remains to be determined if the HBV RNA particles secreted from infected cells contain HBV polymerase, although the presence of the 5′-epsilon sequence suggests that this is likely the case. It is therefore conceivable that spliced RNA could be reverse transcribed and replicated by the polymerase into spliced DNA. Indeed, spliced DNA-containing particles have been detected in the serum of patients with chronic liver diseases and may be responsible for suppressing antiviral immune responses (29, 30, 47). Using single-molecule sequencing analysis, the complexity of these spliced DNA variants was recently reported for a chronically infected patient on nucleos(t)ide analog treatment over a period of 15 years (48). Interestingly, large internal deletions were also present, and the most common variants, Sp1 and Sp3, which contained a spliced intron starting within the core gene and ending in the middle of the S gene, were identical to the most abundant extracellular HBV RNA variants reported in this study. It was further suggested that the deletion of the highly antigenic S region could play a role in mediating immune escape (48). While CAMs could suppress HBV RNA encapsidation and, therefore, the production of spliced RNA and DNA variants, further investigation is needed to understand their potential biological roles in modulating the immune response and maintaining viral persistence.

In summary, we demonstrate that HBV RNA-containing particles are detectable in the supernatant of infected HepaRG cells and PHH, as well as hepatitis B patient sera. Notably, our study is the first to show that extracellular HBV RNA contained pgRNA and also spliced pgRNA variants. The ability of CAMs from different chemical classes to suppress HBV RNA encapsidation, viral replication, and production of HBV RNA- and DNA-containing particles differentiates CAMs from nucleos(t)ide analogs. By blocking multiple steps within the viral life cycle, the antiviral effect of CAMs may intensify viral suppression and can be synergistic with standard therapies, as CAMs and standard therapies have different mechanisms of action. While the serum HBV RNA level may predict the treatment response for patients receiving pegylated interferon and/or nucleos(t)ide analog therapy, the clinical outcome associated with treatment with CAMs inhibiting both HBV DNA and RNA particle production remains to be determined and should be investigated in clinical trials.

## MATERIALS AND METHODS

### Compounds.

NVR 3-1983, BAY 41-4109, and BAY 41-4109-IE were synthesized by WuXi AppTec (Wuhan, China). Lamivudine, tenofovir, and tenofovir disoproxil fumarate were purchased from Toronto Research Chemicals (Toronto, Canada).

### Electron microscopy.

HBV core protein (amino acids 1 to 149) was expressed in Escherichia coli and purified as described previously (12). Electron microscopy of HBV capsid formation was performed as described previously (49). Samples were adsorbed on 200-mesh copper grids coated with a Formvar carbon film, stained with fresh 2% uranyl acetate, and visualized on an FEI Technai T12 transmission electron microscope equipped with a 2K×2K AMT MegaPLUS ES (version 4.0) charge-coupled-device camera.

### Cell culture and HBV infection.

HepG2.2.15 cells (Fox Chase Cancer Center, PA, USA) stably expressing HBV (50) were maintained in Dulbecco's modified Eagle medium containing 10% fetal bovine serum, 380 μg/ml Geneticin, 2 mM l-glutamine, 100 units/ml penicillin, and 10 μg/ml streptomycin. The HBV inoculum was collected from HepG2.2.15 cell supernatants and pelleted by ultracentrifugation through a 20% sucrose cushion. Quantification of HBV in the inoculum was determined by real time-PCR (95°C for 10 min and 45 cycles of 95°C for 10 s and 63°C for 30 s) using the primers 5′-TCT AGA TAC CGC CTC AGC TC (forward) and 5′-GAG TGC AGT ATG GTG AGG TG (reverse) and the probe 5′-TGT ATC GGG AAG CCT TAG AGT CTC CTG. Proliferating HepaRG cells (Biopredics) were maintained using HepaRG medium, which consists of William's medium E, 100 units/ml penicillin, 10 μg/ml streptomycin, 2 mM l-glutamine, and growth supplements (Biopredics). HepaRG cells were differentiated using HepaRG medium containing differentiation supplements (Biopredics) for at least 4 weeks. Cryopreserved primary human hepatocytes (PHH) were purchased from Triangle Research Labs and seeded according to the manufacturer's recommendations (Charlottesville, VA). Differentiated HepaRG cells and PHH were infected with HBV at 100 to 200 genome equivalents per cell in differentiation medium containing 4% polyethylene glycol (PEG) 8000 as previously described (26).

### HBV DNA and RNA quantification assays.

To quantify HBV DNA, supernatants or intracellular lysates from infected cells were treated with Turbo DNase (1 unit) followed by proteinase K (0.5 μg/μl), denatured with 0.2 M NaOH, neutralized with 2 M HEPES, and quantified by a branched DNA assay using probes designed to hybridize to minus-strand HBV DNA (Affymetrix). To quantify intracellular HBV RNA transcripts, infected cells were first solubilized using lysis buffer containing 1% NP-40. To quantify intracellular encapsidated pgRNA, cell lysates were first treated with S7 nuclease (6 units) in lysis buffer containing 10 mM CaCl_2_ to remove cytoplasmic RNA, after which S7 nuclease was inactivated by the addition of EDTA (0.5 M). Total intracellular HBV RNA transcripts, encapsidated HBV RNA, and secreted HBV RNA were each treated with proteinase K prior to RNA quantification by QuantiGene assays using genotype D-specific probes designed to hybridize to the X region of HBV RNA (Affymetrix). To evaluate the specificity of RNA and DNA detection, a viral nucleic acid extraction step was performed on the supernatants of infected HepaRG cells using a ZR viral DNA/RNA kit (Zymo Research). Extracted HBV nucleic acids were then incubated with 1 unit of Turbo DNase (Life Technologies) and 0.5 unit of DNase I (Thermo Fisher) or 25 units of RNase If (New England BioLabs) for 1 h at 37°C. Both DNase and RNase If were inactivated by incubation at 70°C for 20 min, prior to performing quantitation assays as described above.

### HBV antiviral studies.

Differentiated HepaRG cells were seeded in 6-well (2 × 10^6^ cells/well), 12-well (1 × 10^6^ cells/well), 24-well (0.5 × 10^6^ cells/well), or 96-well (6 × 10^4^ cells/well) plates and were infected with HBV at 100 to 200 genome equivalents per cell in the presence of 4% PEG 8000. On day 5 and day 8 postinfection, cells were treated with compounds serially diluted in DMSO and added to HepaRG cell growth medium so that the final DMSO concentration was maintained at 2% across all compound concentrations. Uninfected and infected HepaRG cells not treated with compounds were maintained and replenished with HepaRG cell differentiation medium, which also contained 2% DMSO. After 6 days of incubation with the test compounds, cells and supernatants were collected for HBV DNA and RNA analysis as described above. The levels of secreted HBsAg protein were measured by using an HBsAg chemiluminescence immunoassay kit (Autobio). Cell viability was determined by measuring the amount of intracellular beta-actin RNA using human ACTB hybridization probes and the QuantiGene assay (Affymetrix). In antiviral studies in which cryopreserved PHH were tested, hepatocytes were seeded at 4 × 10^4^ cells/well in 96-well plates and allowed to adhere overnight prior to HBV infection, which was followed by compound treatment and HBV marker analysis as described above.

### HBV cccDNA quantitation assay.

At the end of antiviral compound treatment, infected HepaRG cells were incubated in buffer containing 1% NP-40. The cytoplasmic fraction was removed by centrifugation, and DNA was extracted from the nuclei by using a Quick-DNA universal kit (Zymo Research). As an internal control, human beta-globin DNA was quantitated by real-time PCR (95°C for 10 min and 45 cycles of 95°C for 10 s and 60°C for 30 s) using a TaqMan gene expression assay and a beta-globin primer and probe set (Thermo Fisher). To isolate cccDNA from rcDNA, the extracted DNA was incubated at 37°C for 1 h with T5 exonuclease (New England BioLabs, MA) to degrade the partially double-stranded rcDNA, which contains nicks and gaps. T5 exonuclease was inactivated by the addition of EDTA, and the undigested supercoiled cccDNA was concentrated and purified using a DNA Clean & Concentrator-5 kit (Zymo research). Real-time PCR was performed using FastStart Essential DNA Probes Master (Roche) and the following primers for cccDNA: 5′-CTC CCC GTC TGT GCC TTC T, 5′-GCC CCA AAG CCA CCC AAG, and 5′-FAM/AGG TGA AGC/ZEN/GAA GTG CAC ACG GAC C/3IABkFQ (95°C for 10 min and 45 cycles of 95°C for 10 s, 63°C for 20 s, and 72°C for 30 s), where FAM is 6-carboxyfluorescein, ZEN is internal quencher, and 3IABkFQ is 3′ Iowa black fluorescent quencher. Plasmids containing either the genotype D HBV genome or the human beta-globin gene (Origene) were used to establish standard curves. The levels of cccDNA were normalized to those of the human beta-globin gene.

### Statistical analysis.

Percent inhibition at each compound concentration was calculated by normalization of the signals to the signals from infected HepaRG cells treated with 2% DMSO. In dose-response studies, the compound concentrations were log transformed and inhibition curves were generated by nonlinear fitting using GraphPad software, from which EC_50_ and 50% cytotoxic concentration values were determined. Data points and error bars represent means and standard deviations from at least three independent studies, respectively. Comparisons between data groups were performed using the unpaired *t* test, with *P* values of <0.05 being considered statistically significant.

### Southern blot analysis of HBV DNA within secreted nucleocapsids.

Viral particles from supernatants were resolved on 1% agarose gels. The gel was denatured, neutralized, and transferred to a nylon membrane. Hybridization was performed at 50°C overnight in buffer containing 200 ng a digoxigenin (DIG)-UTP-labeled plus-strand HBV riboprobe. After stringency washes, the membrane was blocked with blocking buffer (wash and block buffer set; Roche) for 30 min. Detection was performed by using anti-DIG-horseradish peroxidase (1:20,000) and a SignalFire Plus enhanced chemiluminescence reagent (Cell Signaling Technology) according to the manufacturer's recommendation and imaged using a ProteinSimple imager.

### HBV RNA analysis by Northern blotting.

Total intracellular HBV RNA was purified using an RNeasy kit with on-column DNA digestion by DNase I according to the manufacturer's recommendations (Qiagen). To isolate intracellular encapsidated HBV RNA, the cytoplasmic extracts from infected cells were treated with S7 nuclease as described above. Viral particles were precipitated with PEG 8000, and pellets were resuspended in buffer containing 10 mM Tris-HCl, pH 8, 100 mM NaCl, and 1 mM EDTA. To prepare secreted HBV RNA, a total of approximately 30 ml of supernatant collected from three wells of a 6-well plate were combined and concentrated 50-fold using 100,000-molecular-weight-cutoff Vivaspin concentrators (GE Healthcare). The concentrated supernatant was treated with 1% NP-40, and capsids were precipitated by PEG 8000. The HBV RNA in the precipitated particles was extracted using the TRIzol LS reagent, precipitated, washed in ethanol, and then resuspended in RNA storage buffer (Ambion). RNA was subjected to electrophoresis through a 1.3% agarose denaturing gel in buffer containing 10 μM aurintricarboxylic acid. UV imaging of ethidium bromide-stained 28S and 18S rRNA was conducted prior to transferring the RNA to a nylon membrane (Nytran SPC; Whatman). Hybridization was performed at 65°C overnight in buffer containing 100 ng DIG-UTP-labeled minus-strand HBV riboprobe. Detection was performed by using anti-DIG-alkaline phosphatase (1:20,000) and the CDP-Star reagent according to the manufacturer's recommendation (Roche) and imaged using the ProteinSimple imager.

### Density gradient ultracentrifugation.

Concentrated culture supernatant from DMSO- or LMV (20 μM)-treated HepaRG cells was layered on linear 10% to 50% OptiPrep density gradient medium (Sigma) and centrifuged at 28,000 rpm (134,000 × *g*) for 2 h at 4°C in a SW41Ti rotor (Beckman Coulter). A total of 20 fractions (0.5 ml) were collected from the top, and the levels of HBV DNA or HBV RNA in each fraction were quantitated as described above.

### Immunoprecipitation of HBV particles.

HepaRG cells were treated or not treated with HBV inhibitors as described above. Supernatants were collected at 11 days postinfection and were precleared with 30 μl protein G beads at 4°C for 3 h. HBsAg antibody (1:500; Abcam) was added to the supernatant, and the mixture was incubated at 4°C overnight. HBV RNA particles were also immunoprecipitated with HBcAg antibody (Dako) in the presence or absence of detergent as previously described (23). Briefly, supernatants were treated with 1% the nonionic detergent NP-40 and 10 mM dithiothreitol at 4°C for 30 min, prior to the 4°C overnight incubation with HBcAg antibodies (1:500). Protein G beads were added to the mixtures, and the mixtures were incubated with constant rocking at 4°C for 4 h. The beads were spun down and washed 5 times in phosphate-buffered saline prior to RNA extraction using the TRIzol LS reagent. Reconstituted RNA from the TRIzol extraction was treated with DNase I for 30 min at 37°C, and the DNase I was inactivated by incubation at 75°C for 15 min, prior to quantification by QuantiGene assays as described above.

### Cloning and sequencing of cDNA transcribed from secreted HBV RNA using patient serum or HepaRG supernatant.

HBV RNA extracted from HepaRG supernatants or patient serum (American Red Cross; samples were approved by the Institutional Review Board at American Red Cross for research purposes) were DNase I treated and reverse transcribed into cDNA using 3′ RACE long primers (24) and a ThermoScript cDNA kit (Invitrogen). Amplification of cDNA was performed by PCR using primers that framed the 5′ and 3′ termini of the HBV genome. The primers used at the 5′ terminus included 5′-CAA CTT TTT CAC CTC TGC CTA (HBV-X long) (24), 5′-ATG CAA CTT TTT CAC CTC TGC (P36), 5′-TCT AGA TAC CGC CTC AGC TC (C-F1), 5′-TTT TGC CTT CTG ACT TCT TTC CTT (C-F2), and 5′-GGA GCT ACT GTG GAG TTA CTC TC (P5); the primers used at the 3′ terminus included 5′-CAA GGT CGG TCG TTG ACA TT (X-R1) and 5′-GCA GAG GTG AAG CGA (X-R2). In addition, a PCR amplification based on overlapping sequences of pgRNA was designed using two forward and reverse primer sets: (i) P5 and 5′-CTG TAA CAC GAG AAG GGG TCC TAG (P14) and (ii) 5′-CTA GGA CCC CTT CTC GTG TTA CAG (P33) and X-R1. Primers P14 and P33 were complementary primers located within spliced introns, as identified from the amplicons obtained using primers that framed the 5′ and 3′ termini of the full-length genome. PCRs were performed using Platinum PCR supermix high fidelity (Thermo Fisher) and cycling conditions of 94°C for 5 min; 40 cycles of 94°C for 30 s, 50°C for 30 s, and 68°C for 3 min; and a final extension at 68°C for 10 min. The PCR amplicons were gel purified and cloned into the PCR4 vector with a TOPO TA Cloning kit (Life Technologies) for sequencing (Genewiz).
